# Manipulation of the microRNA172–*AP2L2* interaction provides precise control of wheat and triticale plant height

**DOI:** 10.1111/pbi.14499

**Published:** 2024-12-11

**Authors:** Chaozhong Zhang, Joshua Hegarty, Mariana Padilla, David M. Tricoli, Jorge Dubcovsky, Juan M. Debernardi

**Affiliations:** ^1^ Department of Plant Sciences University of California Davis CA USA; ^2^ Howard Hughes Medical Institute Chevy Chase MD USA; ^3^ Plant Transformation Facility University of California Davis CA USA

**Keywords:** wheat, triticale, plant height, miRNA172, genome editing, APETALA2

The *REDUCED HEIGHT* (*RHT*) dwarfing alleles *Rht‐B1b* and *Rht‐D1b* were essential in the ‘Green Revolution’. The *RHT1* gene encodes a DELLA protein, which participates in the gibberellin (GA) growth‐stimulating pathway (Peng *et al*., 1999), and truncations of this protein are responsible for the GA‐insensitive semi‐dwarf *Rht1b* alleles (Van De Velde *et al*., 2021). The growth‐repressing effect of *Rht1b* alleles optimized plant height, reduced lodging and improved harvest index, but also reduced above‐ground biomass and coleoptile length, limiting sowing depth and access to deeper soil moisture (Ellis *et al*., 2004). This has triggered the search for GA‐sensitive dwarfing genes with fewer negative pleiotropic effects.

Plant height in grasses is regulated by a complex genetic network, which includes the conserved microRNA172 (miR172)–*APETALA2*‐like (*AP2L*) module (Patil *et al*., 2019; Zhu and Helliwell, 2011). In wheat, miR172 expression is induced during the reproductive transition and regulates flowering time, plant height and both spike and floret development by repressing the expression of *AP2L* genes (Debernardi *et al*., 2017). Reduction of miR172 activity in the semi‐dwarf tetraploid wheat variety ‘Kronos’ (*Rht‐B1b*) using a transgenic target mimicry (MIM172) approach delayed reproductive transition a few days and generated shorter plants with more compact spikes (Debernardi *et al*., 2017).

Among the four *AP2L* genes targeted by miR172 in wheat, *AP2L2* and *AP2L5* regulate flowering transition, stem elongation and spike development (Debernardi *et al*., 2020). Point mutations in the miR172 target site of the *AP2L* genes reduce miR172 activity and generate resistant alleles designated hereafter as *rAp2l*. An *rAp2l*‐*A5* allele originated the domestication gene *Q* and the free‐threshing wheats (Debernardi *et al*., 2017). Additional mutations in the miR172 target site of *Q* or in the homeolog *AP2L‐D5* result in plants with reduced height but, unfortunately, with associated spike defects (Greenwood *et al*., 2017; Zhao *et al*., 2018). In this study, we explore the effects of chemically induced alleles *rAp2l‐A2* from tetraploid and *rAp2l‐B2* from hexaploid wheat (Figure S1a) as well as multiple new CRISPR‐induced alleles. All materials and methods are described in the Materials and Methods in Appendix S1.

The *rAp2l‐A2* EMS‐mutation in the semi‐dwarf Kronos reduced stem length by 21%, whereas the introgression of the *rAp2l‐B2* allele into Kronos or Kronos‐*rAp2l‐A2* backgrounds, reduced stem length by 43–45% (Figure S1a–c, Data S1). We next used CRISPR‐Cas9 with a gRNA specifically targeting the miR172 target site of *AP2L‐B2*, because *AP2L‐A2* has a polymorphism that disrupts the gRNA target (Figure 1a, Figure S2a). We generated multiple independent CRISPR T_0_ events into Kronos (*Rht‐B1b*) and a near‐isogenic tall line (*Rht‐B1a*) (Figure S2, Data S2). Most of the CRISPR mutations were small frameshift indels in the miR172 target site (Figure 1a, Figure S2a), located downstream of the conserved AP2 domains and close to the stop codon (Figure 1a). Both in‐frame and frameshift indels resulted in semi‐dominant dwarfing effects, suggesting that disruptions of the reading frame at the end of the gene have limited effects on AP2L2 activity. The dominance effect of the dwarfing *rAp2l‐B2* alleles was similar in the tall *Rht‐B1a* plants (Figure S2b,c) and the semi‐dwarf *Rht‐B1b* backgrounds (Figure S2d, Data S2).

**Figure 1:**
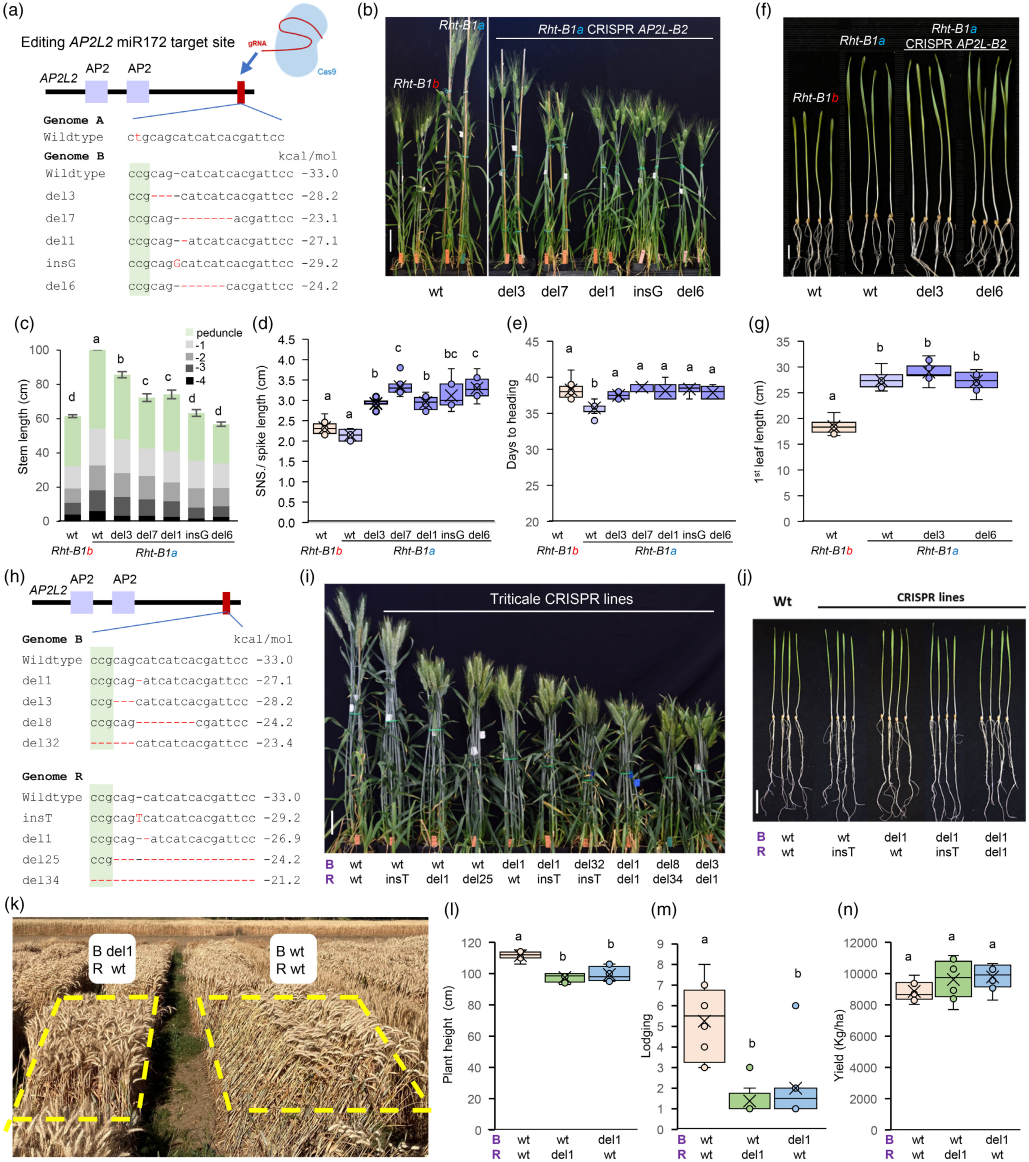
miR172‐resistant *rAp2l2* alleles reduce plant height without affecting coleoptile length or yield. (a) Schematic representation of the *AP2L2* gene indicating the AP2 domains (purple), the miR172 target site (red) and the CRISPR *rAp2l‐B2* alleles (del = deletion, ins = insertion). (b) Kronos *Rht‐B1a* and *Rht‐B1b* plants, and T_2_ *rAp2l‐B2* CRISPR plants 3 weeks after heading, bar = 10 cm. (c) Stem length: internodes are in grey and peduncle in green (*n* = 8). (d) Spikelet density (spikelet number per spike/spike length, *n* = 8). (e) Days to heading (*n* = 8). (f) Seedlings 14 days after germination, bar = 2 cm. (g) Length of the first leaf in 14 days‐old seedlings (*n* = 9–11). (h) *rAp2l‐B2* and *rAp2l‐R2* alleles generated by CRISPR‐Cas9 in triticale variety UC‐Bopak. (i) Triticale wildtype and *rAp2l2* CRISPR plants 3 weeks after heading, bar = 10 cm. Genotypes of *AP2L‐B2* (B) and *AP2L‐R2* (R) homeologs are indicated below each plant. (j) Seedlings 10 days after germination, bar = 1 cm. (k–n) Field experiment comparing triticale wildtype and CRISPR lines (*n* = 8). (l) Plant height. (m) Lodging (1–9 scale, 1 = no lodging and 9 = 100% lodging). (n) Grain yield (kg/ha). Different letters above bars and plots indicate significant differences based on Tukey tests (*P* < 0.05). Estimated interactions energies are to the right of the sequences (kcal/mol). Raw data and statistics are in Data S3.

Independent T_2_ edited lines homozygous for different mutations in both *Rht‐B1a* (Figure 1a–c) and *Rht‐B1b* backgrounds (Figure S3a,b) showed significant effects on plant height that varied depending on the mutations. The strongest *rAp2L‐B2* alleles in the *Rht‐B1a* background reduced plant height to similar levels as *Rht‐B1b* (Figure 1b,c), suggesting that they can be used to replace the *Rht1b* alleles.

The *rAp2l‐B2* plants showed a higher spikelet density (Figure 1d, Figure S3c) as a result of reductions in spike length and slight increases in spikelet number per spike (Data S4). In the *Rht‐B1a* background, the edited lines headed 1.8–2.9 days later, which was comparable to the delay generated by *Rht‐B1b* (Figure 1e). The delay in heading time associated with the *rAp2l‐B2* alleles was slightly stronger in the *Rht‐B1b* sister lines (4.4 to 5.7 days delay, Figure S3d, Data S4). Finally, plants with and without the *rAp2l‐B2* mutations showed similar coleoptile and first‐leaf lengths in both the *Rht‐B1a* (Figure 1f,g, Data S4) and *Rht‐B1b* backgrounds (Figure S3e,f, Data S4). In summary, these results indicate that the *rAp2l‐B2* alleles can be used to reduce plant height with limited pleiotropic effects on spike architecture or heading time, and with beneficial effects in coleoptile length relative to the *Rht1b* alleles.

The highly efficient CRISPR vector makes it possible to rapidly induce different *rAp2l2* dwarfing alleles in elite backgrounds without time‐consuming crosses. To demonstrate this strategy, we generated semi‐dwarf mutants for the triticale cultivar ‘UC‐Bopak’ (PVP 202100269). Triticale is an anthropogenic allohexaploid combining wheat and rye (AABBRR genomes), which delivers significantly high biomass and grain yield (Tamagno *et al*., 2022). However, the taller plant stature of many triticale cultivars combined with their larger and heavier spikes can result in increased lodging. We transformed UC‐Bopak using the same gRNA targeting the miR172 binding site in both *AP2L‐B2* and *AP2L‐R2* homeologs (Figure 1h). Under greenhouse conditions, we observed significant reductions in plant height in edited lines (Figure 1h,i, Figure S4a,b), which were larger in the lines with mutations in both genomes (Data S5). Plant height was correlated with the predicted effect of the mutations on miR172 binding energy both in lines with mutations in *AP2L‐R2* (*R* = −0.94) and in those with mutations in both *AP2L‐B2* and *AP2L‐R2* (*R* = −0.73, Data S5). A combined statistical analysis of the triticale and wheat results (Figure 1a–c and Figure S3a,b) showed that this correlation was highly significant (*P =* 0.0014, Data S5). By selecting different combinations of *rAp2l2* mutations, we were able to fine‐tune triticale plant height (Figure 1i, Figure S4b) without affecting coleoptile and first‐leaf length or heading time (Figure 1j, Figure S4c,e). The edited plants showed more compact spikes but with the same number of spikelets (Figure S4f, Data S5).

Finally, we evaluated lines with 1‐bp deletions in the miR172 target site of *AP2L‐B2* (B) or *AP2L‐R2* (R) under field conditions in two consecutive years. In 2023, we used headrows (Figure S5) and in 2024 small yield plots as experimental units (Figure 1k–n). The plants with the 1‐bp deletions were 17–18 cm shorter the first year (Figure S5a,b) and 12–14 cm shorter the second year (Figure 1l), indicating some interaction with the environment. The spikes of the edited lines were more compact than the wildtype (Figure S5c), but this was not associated with significant differences in grain yield (Figure 1n, Figure S5d). In the second year, the plots of the wildtype variety suffered significantly more lodging than the edited lines (*P* < 0.0001, Figure 1k,m). Although the differences in grain yield were not significant (Figure 1n, Figure S5d), the edited lines showed a combined 9.5% increase in grain yield in the second year (*P =* 0.0528, Data S3), which was likely associated with their superior resistance to lodging.

In summary, we demonstrate that different induced mutations in the miR172 target site of *AP2L2* genes can be used to precisely modulate wheat and triticale plant height. Breeders can use this technology to evaluate multiple plant heights in their top lines without lengthy backcrossing programs. Moreover, the *rAp2l2* alleles did not reduce coleoptile and first‐leaf length, suggesting that they can be a valuable replacement of the gibberellin‐insensitive *Rht1b* alleles.

## Author contributions

CZ performed experiments and contributed to data analyses and manuscript writing. JH contributed to project conceptualization, laboratory and field experiments, data analyses and manuscript writing. MP performed experiments. DMT conducted transformation experiments. JD contributed to project conceptualization, funding acquisition, CZ's supervision, data analyses and manuscript writing. JMD contributed to project conceptualization, performed and designed experiments, analysed data, supervised MP and wrote the original paper draft. JMD and JD generated the final version of the article.

## Conflict of interest

The authors declare no conflict of interests.

## Supporting information


**Figure S1–S5** Supplementary Figures.
**Table S1** Primers used in this study.

**Appendix S1** Supplemental materials and methods.

**Data S1–S6** Supplementary Data.

## Data Availability

Raw data and analyses are provided in the supplemental data (Data S1–S6). Germplasm is available from the authors upon request.
