# Supplementary material for: Manipulation of the microRNA172–AP2L2 interaction provides precise control of wheat and triticale plant height
Source: Plant Biotechnol J. 2024 Dec 11;23(2):333–5. doi: 10.1111/pbi.14499 (PMC11772301; doi:10.1111/pbi.14499)
Supplement: Supplementary file 1 — Figure S1–S5 Supplementary Figures. Table S1 Primers used in this study. [file PBI-23-333-s002.docx]

# Supplementary Figures


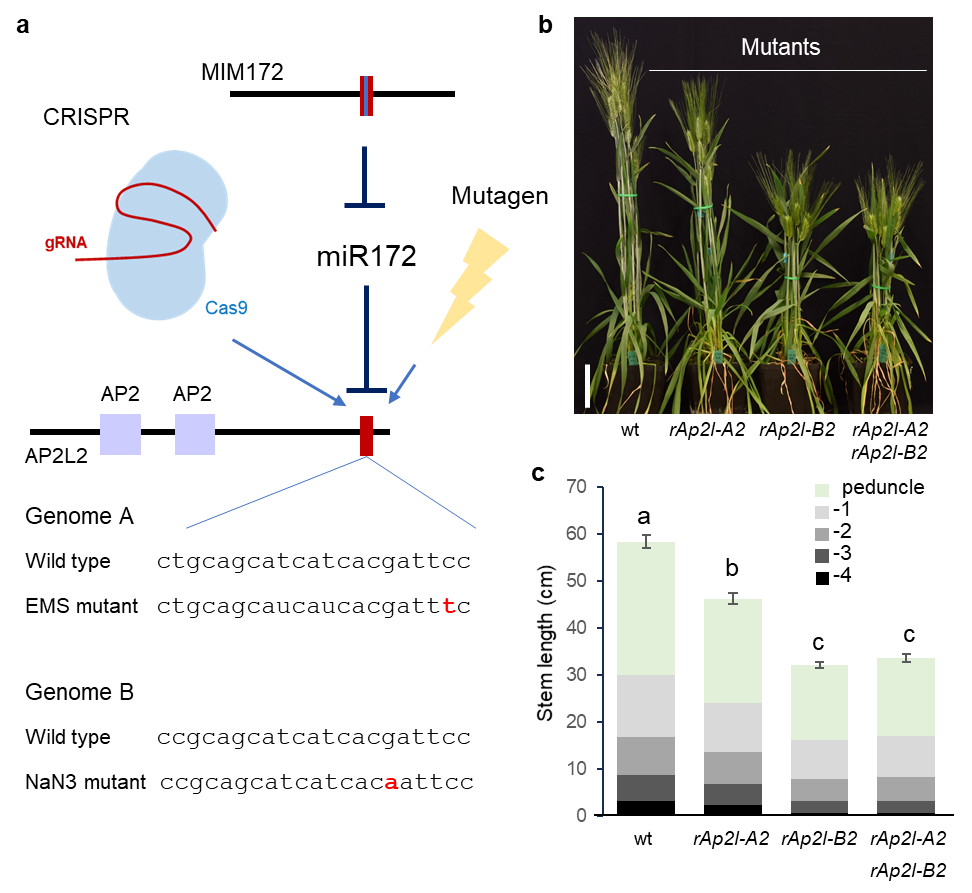


**Figure S1. Reduced miR172 activity on *AP2L2* results in shorter plants. a.** Different approaches to modulate miR172 - *AP2L2* interaction. Mature miR172 activity can be reduced by expressing a target mimicry (MIM172), or by mutating the miR172 target site in *AP2L* genes by CRISPR or chemical mutagenesis. The regions encoding the AP2 domains are indicated in purple and the miR172 target site is in red. Below are the *AP2L-A2* and *AP2L-B2* miR172 target sites in wildtype and *rAp2l2* alleles generated by chemical mutagenesis (in red)*.* **b.** Selected plants three weeks after heading carrying wildtype, *rAp2l-A2*, *rAp2l-B2*, and combined mutant alleles. Bar= 10 cm. **c.** Stem length: internodes are in different gray colors and peduncles are in green (-1 is the closest internode to the peduncle). Different letters above the plots indicate significant differences in total stem length based on Tukey tests (*P*<0.05, n= 16 plants per genotype). These experiments are in a Kronos semi-dwarf background (*Rht-B1b*). Raw data and statistics are available in Data S1.

**
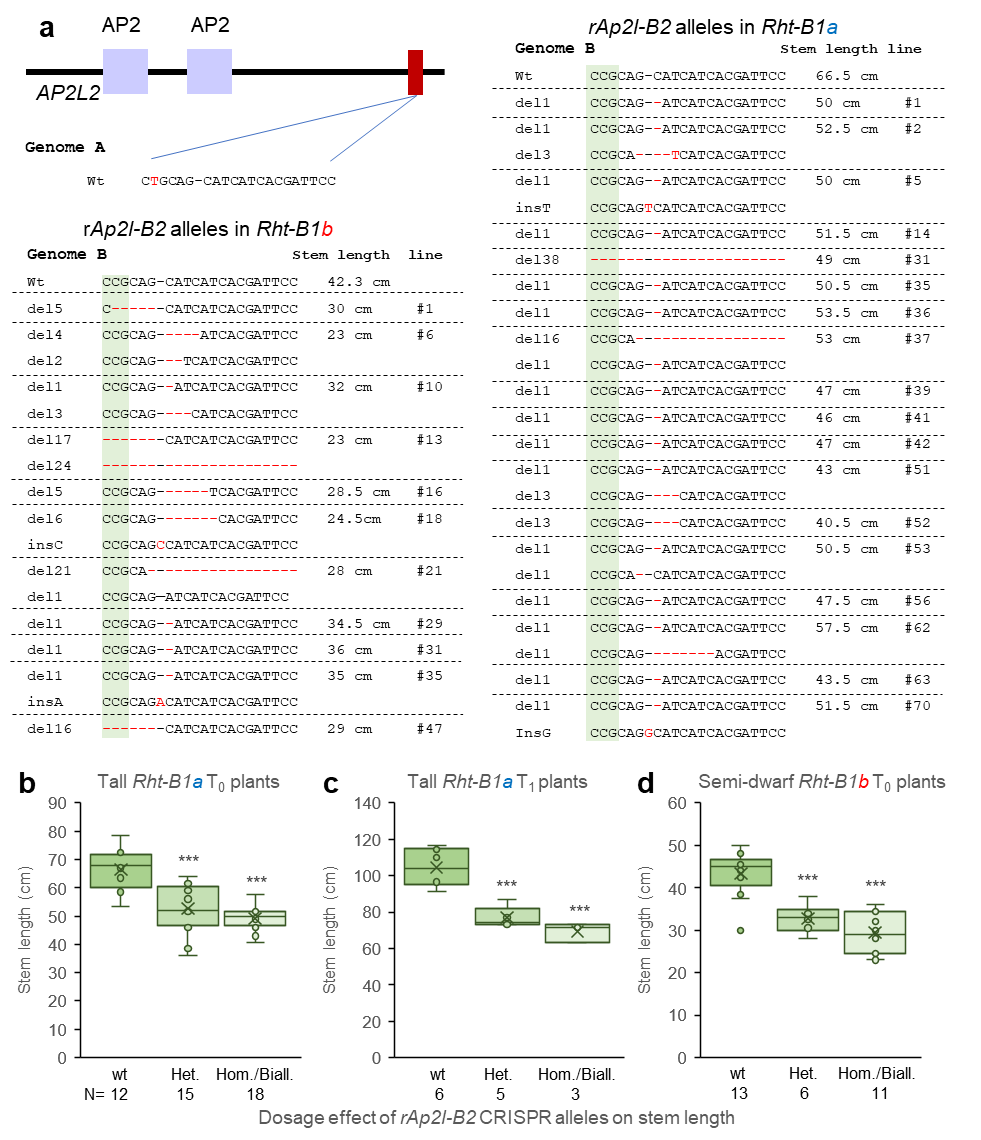
**

**Figure S2. CRISPR induced *rAp2l-B2* alleles show a semidominant dwarfing effect. a.** Schematic representation of the miR172 target sites and different *rAp2l-B2* CRISPR alleles from independent T_0_ lines in semi-dwarf Kronos-*Rht-B1b* and its tall sister line Kronos-*Rht-B1a* (del= deletion, ins= insertion)*.* Stem length is indicated in the right column. (**b-d**) Dosage effect of *rAp2l-B2* CRISPR alleles on stem length (wt= wildtype, Het= heterozygous, Hom./Biall= homozygous or biallelic based). (**b**) Forty-five independent T_0_ events in tall Kronos-*Rht-B1a* grown in small cones. (**c)** Fourteen T_1_ progeny of a T_0_ line heterozygous for del6 in the *Rht-B1a* background grown in larger pots. (**d)** Thirty independent T_0_ lines in the Kronos-*Rht-B1b* background grown in small cones. *** = *P*<0.001 based on Dunnett tests against the wildtype. Numbers below genotypes indicate number of plants tested for each genotype. Raw data and statistics are available in Data S2.

**
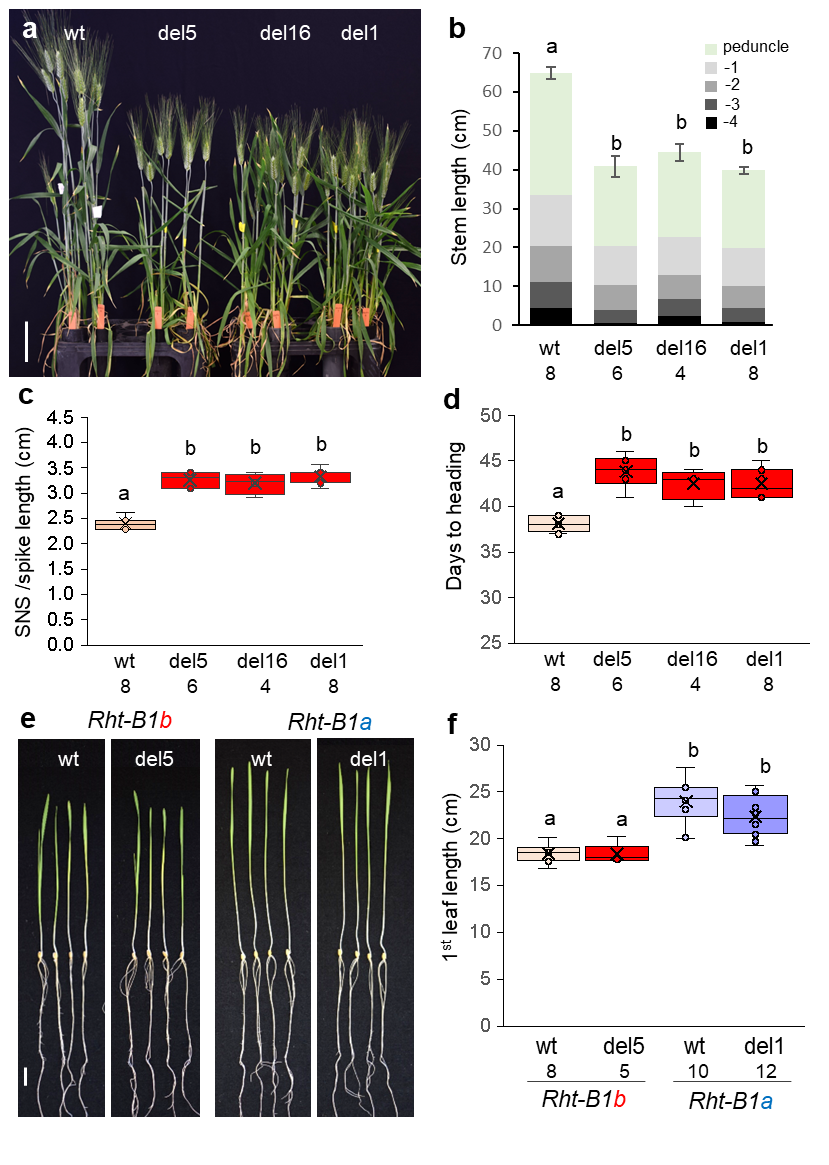
**

**Figure S3. Effect of CRISPR-edits in the miR172 binding site of *AP2L-B2.*** (**a**) Selected Kronos plants after heading carrying the wildtype allele and three independent CRISPR-Cas9 induced deletions. Bar = 10  cm. (**b**) Stem length: internodes are in different gray colors and peduncles are in green (n= 16 plants per genotype). (**c**) Spikelet density calculated as the ratio between spikelet number per spike (SNS) and spike length in cm. (**d**) Days to heading. **a** to **d** in Kronos-*Rht-B1b* background. (**e**) Seedlings of wildtype (wt) and edited lines in *Rht-B1b* and *Rht-B1a* backgrounds. Bar = 2 cm. (**f**) Length of the first leaf. Note the shorter seedlings in *Rht-B1b* relative to *Rht-B1a.* Different letters above the bars or box-plots indicate significant differences based on Tukey tests (*P*<0.05). Numbers below the genotypes indicate the number of plants measured per genotype. Raw data and statistics (including coleoptile length) are available in Data S4.


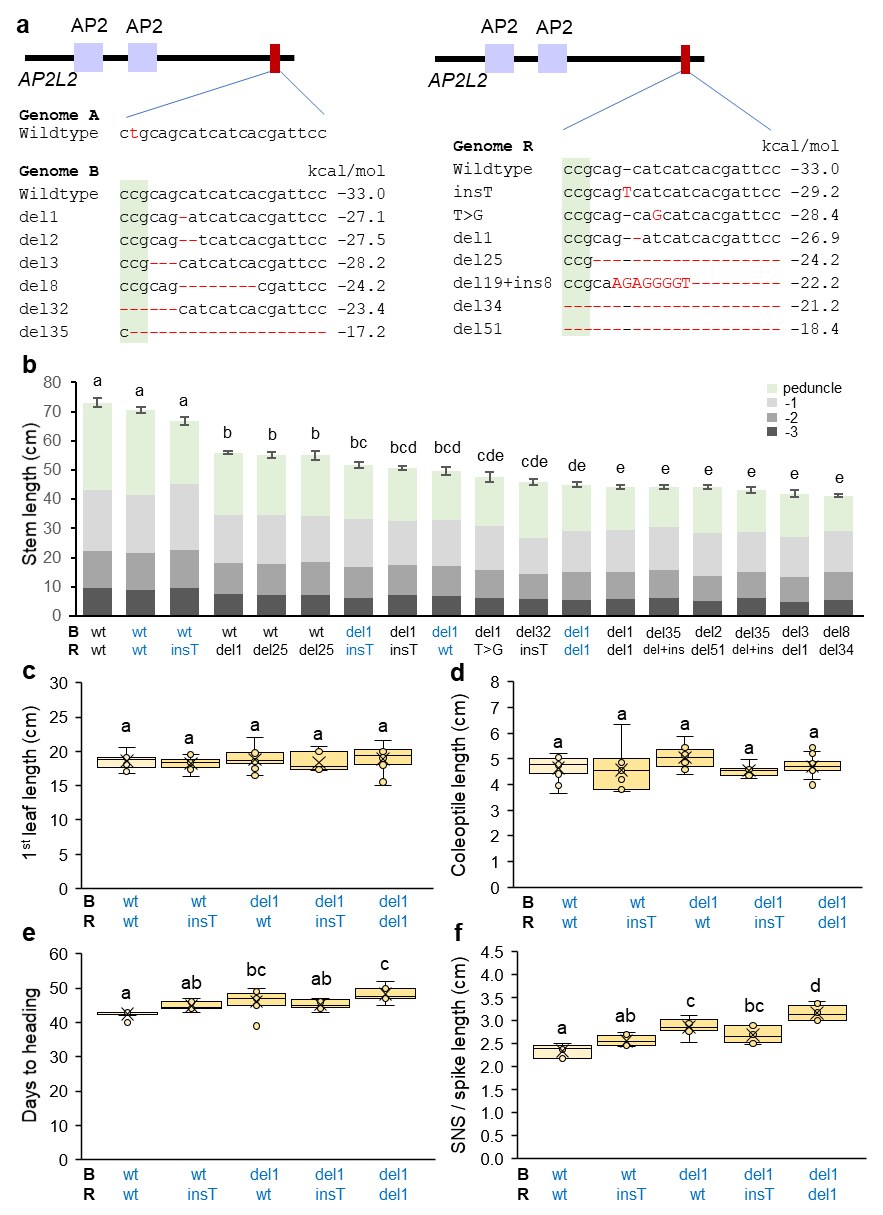


**Figure S4. *rAp2l-B2* and *rAp2l-R2* alleles modulate plant height in triticale cultivar UC-Bopak. a.** Schematic representation of *AP2L2* and sequences of the CRISPR-edited sites in the miR172 target sites in *AP2L-B2* (B genome from wheat) and *AP2L-R2* (R genome from rye). del= deletion, ins= insertion*.* The estimated interaction energy in kcal/mol is indicated to the right. **b.** Stem length in selected lines: internodes are in different grey colors and peduncles are in green (n=8 except wt/del25 n=6). The *AP2L2* genotypes of the B and R genomes are indicate below each bar. Genotypes in blue are the same in panels **b** to **f**. (**c**) Length of first leaf in 10 days-old seedlings. (**d**) Coleoptile length in cm. (**e**) Days to heading. (**f**) Spikelet density (spikelet number per spike / spike length in cm). **c-f** n=8. Different letters above bars and plots indicate significant differences based on Tukey tests (*P*<0.05). Raw data and statistics are available in Data S5.


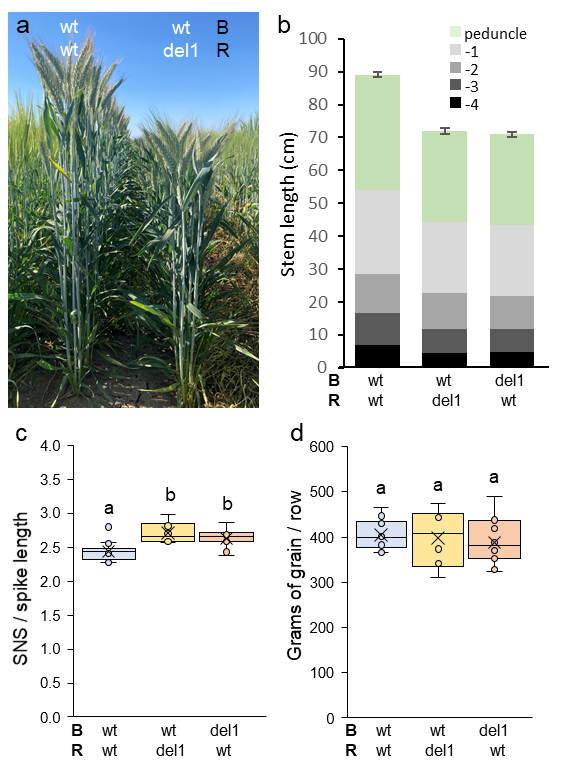


**Figure S5.** Field experiment (2023 field season) comparing the triticale variety UC-Bopak and CRISPR lines with 1-bp deletions in the miR172 binding site of *AP2L-B2* (wheat B genome) or *AP2L-R2* (rye R genome). **a.** Selected rows comparing UC-Bopak with a CRISPR-edited line carrying the 1-bp deletion in *AP2L-R2*.  **b.** Stem length: internodes are in different gray colors and peduncles are in green. **c.** Spikelet density (spikelet number per spike / spike length in cm). **d**. Grams of grains produced per row. wt/wt= 14 headrows, wt/del1= 6 headrows, del1/wt= 15 headrows. Headrows were used as experimental unites in a completely randomized design. Four to six plants (subsamples) were measured per row and values were averaged for the statistical analyses. Different letters above the bars and box-plots indicate significant differences based on Tukey tests (*P*<0.05). Raw data and statistics are available in Data S6.

# Supplementary Table

Table S1. Primers used in this study.

| **Primer Name** | **Sequence** | | **Purpose** |
| --- | --- | --- | --- |
| JD633_CRISPR-seq-Fw | TCCTCTGTCACGGAAGCGTTGTAAAACGACGGCCAGTG | Detection of T-DNA insertion | |
| JD633_CRISPR-seq-Rev | TTTAGCCTCCCCACCGACtccagcagagttctgaccg | Detection of T-DNA insertion | |
| ZCZp166_zCas9-F1 | AGACCGTGAAGGTTGTGGACGAGCT | Detection of T-DNA insertion (CAS9) | |
| ZCZp201_TaCAS9-R | CTTAACCTCCCTGATCAGCTTGTCGT | Detection of T-DNA insertion (CAS9) | |
| qPCR1_GIF1-Rev2 | GGTGCCCTTGAGGTACTCC | Detection of T-DNA insertion (GIF1) | |
| qPCR1_GIF1-Fw2 | AACCAAGCTAAGCTCCAGCA | Detection of T-DNA insertion (GIF1) | |
| Hpt.miki.F | GGCCTCCAGAAGAAGATGTTGG | Detection of T-DNA insertion (Hpt) | |
| Hpt.miki.R | GAGCCTGACCTATTGCATCTCC | Detection of T-DNA insertion (Hpt) | |
| ZCZp310_Fw-AP2-B/R | TCCTCTGTCACGGAAGCGAYGGCTGGGGCAACGTCGTC | amplicon seq of Ap2L2-R | |
| ZCZp464_AP2L2-B-Fw | TCCTCTGTCACGGAAGCGCACGAGCTGGCAGCCGCCC | amplicon seq of Ap2L2-B/R | |
| ZCZp296_Rev-AP2-2Crisp | TTTAGCCTCCCCACCGACCCGTTCTTCTGGAGCCAGCT | amplicon seq of Ap2L2-B/R | |
| ZCZp447_AP2L2-A-FAM-T | GAAGGTGACCAAGTTCATGCTgcTgcagcatcatcacgattT | KASP for *rAp2L-A2* | |
| ZCZp448_AP2L2-A-VIC-C | GAAGGTCGGAGTCAACGGATTgcTgcagcatcatcacgattC | KASP for *rAp2L-A2* | |
| ZCZp450_AP2L2-A-Common2 | tGgCAAgCtTAGGtGGgTcT | KASP for *rAp2L-A2* | |
| ZCZp486_TaAP2-B-FAM-Wt | GAAGGTGACCAAGTTCATGCTAGCAGCCGCAGCATCATCACG | KASP for *rAp2L-B2* | |
| ZCZp487_TaAP2-B-VIC-Mut | GAAGGTCGGAGTCAACGGATTAGCAGCCGCAGCATCATCACA | KASP for *rAp2L-B2* | |
| ZCZp488_TaAP2B-common | ACGGATGGTCTCCGGGTAC | KASP for *rAp2L-B2* | |
