# Supplementary material for: Manipulation of the microRNA172–AP2L2 interaction provides precise control of wheat and triticale plant height
Source: Plant Biotechnol J. 2024 Dec 11;23(2):333–5. doi: 10.1111/pbi.14499 (PMC11772301; doi:10.1111/pbi.14499)
Supplement: Supplementary file 2 — Appendix S1 Supplemental materials and methods. [file PBI-23-333-s003.docx]

**Supplemental Materials and Methods**

**Plant material and growing conditions.**

The tetraploid wheat variety Kronos used in this study has the *AP2L-5A* Q allele, which confers the subcompact spike phenotype and free-threshing character, and the *Rht-B1b* allele that confers the semi-dwarf phenotype. We also use a taller near-isogenic line of Kronos that was previously generated by introgressing the *Rht-B1a* allele from the tetraploid wheat cultivar ‘Gredho’ (PI 532239) into Kronos through six rounds of backcrossing (Kronos-*Rht-B1a*) ^1^. A KASP marker to differentiate the two *Rht-B1* alleles was also developed in the previous study ^1^.

Chemically induced mutants in the miR172 target site of the *AP2L2* genes were also described previously ^2^. These mutations reduce the ability of miR172 to cleave the *AP2L2* mRNAs and are designated as resistant alleles or *rAp2l2* alleles. The *rAp2l-A2* allele was previously generated in Kronos (mutant K2236) by ethyl methanesulfonate (EMS) ^2^, whereas the *rAp2l-B2* allele was generated with sodium azide in the winter hexaploid variety Wedgetail (*Rht-B1a* and *Rht-D1b*), which was mutagenized using sodium azide ^3^.

For this study, we combined the *rAp2l-A2* and *rAp2l-B2* mutations in Kronos by crossing the mutant lines Kronos-*rAp2l-A2* and Wedgetail-*rAp2l-B2*. As Wedgetail-*rAp2l-B2* is a winter cultivar, we vernalized it for seven weeks to induce flowering. The F_1_ plants were pollinated with Kronos-*rAp2l-A2*, and the resulting BC_1_F_1_ plants heterozygous for *rAp2l-A2* and *rAp2l-B2* were backcrossed to Kronos-*Rht-B1b* two more times. The BC_3_F_2_ population was genotyped using KAPS markers for *rAp2l-A2* and *rAp2l-B2* (primers in Table S1), and the four possible homozygous combinations were selected (wt, *rAp2l-A2*, *rAp2l-B2*, and *rAp2l-A2* *rAp2l-B2*).

The commercially available triticale (× *Triticosecale*) cultivar used for transformation is a hexaploid (genomes AABBRR) spring cultivar from the UC Davis breeding program, released as ‘**UC-Bopak’ (**PVP 202100269). UC-Bopak is ~110 cm tall and has very high grain yields relative to other triticales grown in the region.

Multiple independent transgenic plants were generated in Kronos, Kronos-*Rht-B1a* and UC-Bopak (see transformation and selection methods below). Transgenic plants in the T_0_ generation were genotyped by amplicon sequencing to identify edits in the miR172 target site of *AP2L2* genes. Selected T_0_ lines with edits in the target region were advanced to T_2_ in Kronos and to T_5_ in triticale. Plants were genotyped by amplicon sequencing to confirm the edits, and the presence of T-DNA sequences carrying the CRISPR-Cas9 was determined by PCR using primers described in Table S1. For triticale, we also crossed T_1_ plants with the wildtype **UC-Bopak**, and advanced the progeny to the F_3_ generation, where we selected transgene-free edited plants. Field experiments were performed in 2023 with F_4_ or T_5_ plants and in 2024 with F_5_ or T_6_ plants.

The transgenic plants were grown in cones in PGR15 growth chambers (Conviron, http://www.conviron.com) adjusted to 16 h of light (22°C) and 8 h of darkness (18°C). The intensity of the sodium halide lights measured at the height of plant heads was ~260 μM m^-2^s^-1^.

For phenotypic evaluation, mutants and transgenic plants were grown in a greenhouse under long-day photoperiod (16-h light / 8-h dark, natural light supplemented with artificial light for 16 h). Temperatures oscillated between 23 °C during the day and 20 °C during the night. Plants were germinated in Petri dishes at 4°C for 3–5 days. After the first leaf emerged, we transplanted the seedlings into one-gallon pots (two plants each pot), and recorded days to heading from this day until emergency of half of the main spike from the flag leaf. Spikelet number per spike, spike length, and stem length (internodes and peduncle) measurements were taken at maturity.

**Field experiments**

The CRISPR-edited lines of the triticale variety UC-Bopak with 1-bp deletions in the miR172 binding site of *AP2L-B2* (B del1) or *AP2L-R2* (R del1) and a sibling line without edits (wt) were evaluated in field experiments over two seasons at the UC Experimental Field Station in Davis, CA (38° 32′ N, 121° 46′ W). During both field seasons plants received a total of 225 kg/ha of N applied as ammonium sulfate, and irrigation and herbicide applications when needed. The 2023 field experiment was sown in January 2023 and harvested in June 2023. This experiment was organized in a completely randomized design using 2-m long rows as experimental units (~100 grains per row) and consisted of 14 replications of wt, 15 replications of B del1 and 6 replications of R del1. Seeds harvested from these rows were used in field experiments in the 2024 field season that was sown in December 2023 and harvested in June 2024. The 2024 experiment included 8 replications for each genotype and was arranged in a completely randomized design, using small plots as experimental units (4.5 m x 1.4 m) with a seeding density of 262 grains per m^2^.

In both field seasons, we measured total plant height from the soil to the top of the main spike excluding awns. In the 2023 season, we randomly selected 4 to 6 primary tillers from each row, and obtained the length of peduncles, internodes, and spikes (in cm). We also determined the spikelet number per spike (SNS) and calculated spikelet density as SNS/spike length. Measurements from the 4-6 subsamples were averaged, and row means were used in the statistical analyses. We also determined grain yield per row (in grams) for the 2023 experiment. In the 2024 season, yield plots were evaluated for lodging on a 1-9 scale, where 1 indicates no lodging, 5 indicates more than half of the plot is lodged by 45 degrees or more, and 9 indicates the entire plot was flat on the ground. Grain from each yield plot was harvested with a Zurn 150 plot combine and yield was calculated based on measured plot length and converted to kg of grain per hectare.

**CRISPR vector and transformation**

The miR172 target sites of the *AP2L-B2* in Kronos and both *AP2L-B2* and *AP2L-R2* in UC-Bopak were edited using CRISPR-Cas9. We designed a gRNA that specifically targets the miR172 sequence in these two homeologs (Fig. 1a, h). The gRNA was cloned into the binary vector JD633 that includes Cas9 and GRF4-GIF1 cassettes ^4^ (Addgene Plasmid #160393), and the final vector was transformed into the *Agrobacterium* strain EHA105. Transgenic plants were generated at the UC Davis Plant Transformation Facility (<http://ucdptf.ucdavis.edu/>). Immature embryos from Kronos-*Rht-B1a*, Kronos-*Rht-B1b* and triticale were inoculated with *Agrobacterium*. Transgenic plant selection was done using hygromycin, and transgene insertion was validated by DNA extraction and PCR.

**CRISPR Genotyping by amplicon sequencing**

We collected leaf samples from plants and isolated genomic DNA using the commercial DNeasy kit (Qiagen), according to the manufacturer’s protocol. The detection of genome editing events was done by amplicon sequencing as described previously ^5^. Briefly, we performed polymerase chain reactions (PCRs) with primers flanking the regions targeted by the different gRNAs (primers in Table S1). Then, we added barcoded adaptors through a second nested PCR, pooled the PCR products, purified, and subjected them to CRISPR sequencing using the sequencing services provided by MGH CCIB DNA Core (<https://dnacore.mgh.harvard.edu/new-cgi-bin/site/pages/crispr_sequencing_main.jsp>)..

**Statistical analyses**

The raw data and statistical analyses supporting the figures and supplemental figures in this study are provided in the Supplementary Data (excel file). Single way ANOVAS were performed for every trait. Homogeneity of variances were tested using Levene’s test and normality of residuals using the Shapiro-Wilk test. When ANOVA assumptions were not met, data was transformed using power transformations. In a few cases, when we could not find any appropriate transformation to satisfy both ANOVA assumptions, we used pairwise non-parametric Kruskal-Wallis tests. All statistical analyses were performed using SAS version 9.4.

Distribution of the data is presented using box-plots except for stem length where bar graphs were used to present the individual contributions of the peduncle and internodes to the total stem length. In the box-plots (generated with Excel), the middle line of the box represents the median and the x represents the mean. The bottom line of the box represents the first quartile and the top line the third quartile. The whiskers extend from the ends of the box to the minimum and maximum values.

To calculate the degree of dominance of the *rAp2l2* alleles, we genotyped segregating T_0_ and T_1_ lines in the *Rht-B1a* and *Rht-B1b* backgrounds and classified the plants as heterozygous, homozygous wildtype or homozygous/biallelic mutant. Degree of dominance was calculated as the difference between the average stem length of the heterozygotes and the midpoint value between the homozygotes, divided by the additive effect ^6^.

**Supplemental Methods References**

1. Zhang, J. *et al.* *Proc. Natl. Acad. Sci. USA* **120**, e2300203120 (2023).

2. Debernardi, J.M., Greenwood, J.R., Jean Finnegan, E., Jernstedt, J. & Dubcovsky, J. *Plant J.* **101**, 171-187 (2020).

3. Chandler, P.M. & Harding, C.A. *J. Exp. Bot.* **64**, 1603-1613 (2013).

4. Debernardi, J.M. *et al.* *Nat. Biotechnol.* **38**, 1274-1279 (2020).

5. Zhang, J. *Check CRISPR editing events in transgenic wheat with next-generation sequencing,* (Springer US, New York, NY, 2022).

6. Falconer, D.S. *Introduction to quantitative genetics.,* ( Oliver & Boyd Edinburgh, 1964).
